# Public Hygiene—Resolutions and Suggestions

**Published:** 1897-06

**Authors:** W. Neal Watt

**Affiliations:** Austin, Texas


For the Texas Medical Journal.

### PUBLIC HYGIENE—RESOLUTIONS AND SUGGESTIONS.

BY W. NEAL WATT, M. D., AUSTIN, TEXAS.

[Read at the meeting of the Austin District Medical Association, March 21, 1897.]

SINCE the importance of sanitary science in its relations to a community can hardly be estimated, and in consideration of the fact more and more consideration is being attached to the preservation of the public health and the prevention of disease (as well as its cure) by modern medicine, as time rolls on, and inasmuch as our fair city is without adequate and systematic protection of this kind; be it

*Resolved*, That it is the sense of this body that some action should be taken at once by the proper authorities to establish a health department on proper lines and in accordance with sanitary laws and regulations. We would suggest that since the duties of a sanitary inspector involve such subjects as the natural and acquired features of the locality; its meteorological peculiarities; the social and sanitary state of the population; the character of the soil, ground waters, wells and springs; the water supply; plans of drainage and sewerage; the distribution of buildings and open spaces, whether paved or unpaved; the sanitary arrangement of houses, especially those of the poorer classes; the management of burial grounds and the arrangements for the burial of the dead; the nature of the manufacturing and other industrial establishments; the housing of the poor and the facilities for bathing, washing, etc.; the conduct of slaughtering houses and all establishments where foods are prepared; the examination of foods with regard to their wholesomness; the sanitary inspection of schools and school children; the regulations for cleansing the public ways and markets; the removal and disposal of domestic and trade refuse; the examination of persons and houses with the object of restricting and suppressing contagious and infectious diseases of local origin, and of vessels and passenger trains in order to prevent the introduction of such diseases from without; and lastly, but not least, the establishment of a record of vital statistics. A man qualfied for the work in hand, a medical man, should be placed at the head of the public health department, with exclusive control of the sanitary inspection and all matters appertaining to this department.

It would be better to have one man in charge than a number of men, as provided by a board of health, since boards of health are unwieldy and expensive factors; delaying action and involving great expense, without corresponding advantages, the single health officer being just as efficient without the objections named. This head of the sanitary department, whose title would be Sanitary Inspector, should be graduated in medicine of a college of standing and repute, and possess the necessary outfit and paraphernalia, including a first-class, high power microscope, and a knowledge of its manipulation. We find that some of the cities have these aforesaid boards of health, composed usually of the mayor as an ex-officio member, the city physician and two resident physicians. Others relegate this work to the city physician, thus conferring duties of a preventive kind, as well as those of a curative kind, but in these cities there is no duality of office, the cities having their medical representatives and the counties theirs. In consideration of the fact that the duties of this office are multiform and various, receiving the larger portion of the physician's time for their proper and thorough performance, it would be well to select a physician other than the city physician, in as much as this officer's time is about taken up by his dual office, acting as he is as the medical representative of the city, and as that of the county also. This individual, immediately after his election to the office in question, should select for enactment into law such articles as he might deem proper for his guidance and the efficient administration of his office, said articles to be entitled, "The Sanitary Laws of the City of Austin," and to contain provisions for their enforcement and promulgation. We deeply deplore the inefficiency and irregularity of the present apology for public hygiene, and the fact that a layman is at the head of a department manifestly professional; especially humiliating it is to us when we consider the fact that the duties assumed are medical in their scope and meaning. Imagine a consultation between State Health Officer Swearingen and City Health Officer (so-called) Calhoun on a question of inland quarantine, involving disease and disease technicalities, an incongruity, indeed; it would be laughable if the subject were not too serious to laugh about. Health and life are involved, as well as the reputation and happiness of this capital city. It is a subject of higher import than one of mere dollars and cents, coming, as it does, so close to the chief boons of our existence—health and longevity. Public Health is a subject of comparatively recent birth, so far as organization and legislation are concerned, any way, and there has been much confusion in arranging departments of this kind. We are sorry to say we are still among the confused, and that it is high time we are correcting our position, as other cities have already done.

